# Ferroptosis-new avenues for the treatment of multidrug-resistant bacteria: A review

**DOI:** 10.1097/MD.0000000000045002

**Published:** 2025-10-17

**Authors:** Yonglang Chen, Xiaohui Bi, Gang Fu, Lüwei Wen

**Affiliations:** a Department of Laboratory Medicine, South China Hospital Affiliated to Shenzhen University, Shenzhen, China.

**Keywords:** antibacterial therapy, ferroptosis, multidrug resistance, nanotechnology

## Abstract

The rise of multidrug-resistant bacteria presents a critical global health threat, demanding innovative therapeutic approaches. This review highlights the key findings on ferroptosis – a novel iron-dependent cell death pathway – as a promising strategy for combating bacterial infections and resistance. Central to ferroptosis are iron-mediated lipid peroxidation and reactive oxygen species (ROS) accumulation, which exert antimicrobial effects through 2 primary mechanisms: host cell ferroptosis triggering lipid peroxidation and direct ferroptosis-like bacterial death induced by ferrous iron.

Lizeng Gao demonstrate that nanotechnology can enhance ferroptosis-based therapy by enabling targeted drug delivery, minimizing off-target effects, and improving efficacy against resistant bacteria. Additionally, polysulfides amplify ferroptotic activity by suppressing bacterial antioxidant defenses, reducing the need for high doses of ferroptosis inducers. Compared to conventional antibiotics, ferroptosis offers distinct advantages in overcoming resistance mechanisms.

However, challenges remain, including the complexity of iron metabolism and potential toxicity, which must be addressed for clinical translation. Future research should focus on elucidating ferroptosis mechanisms and advancing delivery technologies, such as nanoparticle-based systems, to optimize precision and efficacy in vivo. These insights position ferroptosis as a viable alternative for treating resistant infections, pending further refinement.

## 1. Introduction

The proliferation of antibiotic resistance (AR) among bacteria has been identified by the World Health Organization (WHO) and global health agencies as a principal threat to public health, particularly in regions where healthcare resources are limited.^[1]^ The spread of drug-resistant bacteria not only leads to treatment failures but also exacerbates the disease burden, prolongs hospital stays, escalates treatment costs, and increases mortality rates.^[2]^ The impending “post-antibiotic era” raises the specter of once-tractable bacterial infections becoming life-threatening again.^[3]^ Despite the discovery of new antibiotics in the mid-20th century, the pace of development has slowed significantly due to the high costs and low market returns associated with research and development.^[4]^ This challenge has prompted the scientific community to explore innovative anti-infection strategies, such as immunotherapy, phage therapy, and interventions based on novel cellular death mechanisms, including ferroptosis.^[5]^ As an innovative form of regulated cell death, ferroptosis, when combined with state-of-the-art technologies like nanotechnology, presents a non-antibiotic approach to combat drug-resistant bacterial infections.^[6]^ This review synthesizes the mechanisms of iron regulation in humans and bacteria, elucidates the principles of applying ferroptosis, assesses the current research on the combination of ferroptosis with nanotechnology, and discusses the advantages and challenges of using ferroptosis in treating multidrug-resistant bacteria. It provides a theoretical foundation for the development of new treatment pathways against multidrug-resistant bacteria.

## 2. The role of iron in the human body and bacteria

Iron is an essential element within the human body, playing a crucial role in oxygen transport, energy metabolism, immune function, and neurotransmitter synthesis. The primary sources of iron include heme-binding proteins and iron ion-binding proteins. An increase in the concentration of ferrous ions within the human body can readily lead to iron poisoning.^[7]^ The hazards associated with iron overload encompass a spectrum of diseases, including liver cirrhosis, myocardial damage, diabetes, neurodegenerative diseases, gastrointestinal damage, immune system suppression, and skin pigmentation, all stemming from the excessive burden of iron.^[8]^

In bacteria, iron serves as a central catalyst in numerous biological processes, being essential for their physiological activities and survival. Initially, iron acts as a cofactor for numerous key enzymes involved in bacterial redox reactions, facilitating electron transfer within the electron transport chain, which is particularly crucial for bacterial energy generation under both aerobic and anaerobic conditions.^[9]^ Moreover, iron engages in pivotal metabolic processes such as oxidative phosphorylation and fermentation through its active forms – ferric iron (Fe³^+^) and ferrous iron (Fe²^+^) – during the conversion process.^[10]^ Iron significantly impacts DNA synthesis and repair; during the rapid growth and division of bacteria, the demand for iron escalates, interplaying with enzymes involved in DNA synthesis, such as ribonucleotide reductase.^[11]^ Consequently, iron deficiency directly impedes DNA synthesis, thereby inhibiting bacterial growth. Iron is also a critical component in maintaining the structure and function of vital intracellular constituents. It ensures structural integrity and smooth biosynthetic function in ribosome assembly and protein synthesis.^[11]^ Furthermore, iron is implicated in the formation and stability of bacterial biofilms, which is vital for bacteria to withstand immune defenses and antibiotic assaults, safeguarding bacteria from external pressures.^[12]^ When the iron content within bacteria is excessive, the surplus iron can generate hydroxyl radicals through the Fenton reaction, a chemical reaction wherein iron ions – predominantly ferrous iron Fe²^+^ – produce hydroxyl radicals in the presence of hydrogen peroxide (H_2_O_2_).^[13]^ Hydroxyl radicals are highly reactive and destructive molecules that rapidly interact with surrounding biomolecules, inflicting oxidative damage upon them.^[14]^ Additionally, excessive iron can activate a suite of stress responses, including the expression of iron chelation proteins or antioxidant enzymes, to neutralize or mitigate the damage caused by iron overload.^[15]^ However, when the defense mechanisms are insufficient to fully counteract oxidative stress, it can result in restricted bacterial growth or even death. The Fur protein, as the bacterial iron-sensing system, can sense the intracellular iron concentration and modulate the expression of iron-related genes. Under iron-deficient conditions, Fur activates iron absorption genes, whereas under iron excess, it represses iron uptake.^[16]^ Moreover, bacterial virulence factors are highly dependent on iron, encompassing toxin synthesis and secretion, enzyme activity, adhesion factor synthesis, biofilm formation, metabolic regulation, virulence enhancement, and gene regulation.^[17–20]^

## 3. The “iron competition war” between the human body and bacteria

Nutritional immunity refers to the innate immune strategy employed by hosts to regulate the availability of key nutritional elements within the body, thereby restricting the growth and proliferation of pathogens. This mechanism aims to control the essential metal ions required for pathogen survival, thereby inhibiting their infectious capabilities.^[21]^ Iron, being one of the essential trace elements for the growth of both hosts and pathogens, is subject to a host-mediated restriction strategy when facing infection. This iron restriction strategy, known as “iron withholding,” is one of the core mechanisms of nutritional immunity.^[22]^ This includes the regulation of hepcidin: during bacterial infection, the host upregulates the expression of hepcidin, which inhibits the function of the iron transporter ferroportin, reducing iron absorption in the intestine and the release of iron from the reticuloendothelial system. This lowers the concentration of free iron in the serum, effectively blocking the pathogen’s access to iron and restricting its growth.^[23]^

The concentration of free iron ions in the human body is several times lower than the iron ion concentration required for bacterial growth.^[24]^ To cope with the scarcity of iron in the host environment and the limitations of nutritional immunity, bacteria have evolved multiple strategies to acquire iron, including siderophore pathways, direct receptor-mediated uptake, iron reduction systems, and competitive inhibition. These iron-acquisition strategies allow bacteria to survive and reproduce in the iron-deprived host environment.^[12,15,25,26]^ However, these mechanisms also become targets of the host’s defense system. By regulating iron metabolic pathways, the host can restrict the growth and spread of pathogens, forming a continuous “iron competition war” between the host and pathogens.^[27]^ It is worth mentioning that researchers have developed a strategy of combining antibiotics with siderophores. This leverages the bacteria’s craving for iron to more effectively deliver antibiotics into the bacteria, thereby enhancing the drug’s antibacterial effect.^[28]^

## 4. Ferroptosis and its regulatory mechanisms

Ferroptosis is a distinct form of regulated cell death initially proposed by Dixon and colleagues in 2012, characterized by iron-dependent lipid peroxidation and the accumulation of reactive oxygen species (ROS).^[29]^ Unlike other mechanisms of cell death such as apoptosis, autophagy, and necrosis, ferroptosis primarily relies on iron-catalyzed Fenton reactions and the resulting lipid peroxidation processes.^[30]^

The main regulatory mechanisms of ferroptosis involve iron metabolism, lipid metabolism, and antioxidant defense systems.^[31]^ Iron enters cells through pathways such as transferrin (Tf) and ferritinophagy, and excessive iron generates ROS through Fenton reactions, driving lipid peroxidation.^[32]^ Polyunsaturated fatty acids (PUFAs) are the primary targets of lipid peroxidation, especially PUFAs in phosphatidylethanolamine.^[33]^ Key enzymes in lipid metabolism, such as ACSL4 and ALOX, play crucial roles in regulating the oxidation of PUFAs.^[29]^ Antioxidant defense mechanisms, such as glutathione peroxidase 4 (GPX4), effectively prevent the occurrence of ferroptosis by inhibiting lipid peroxidation.^[34]^

## 5. The mechanisms of ferroptosis in achieving bacterial inhibition and ferroptosis inducers

### 5.1. Ferroptosis in macrophages

Macrophages infected with bacteria significantly increase intracellular iron levels through 3 main pathways: the NCOA4 ferritin degradation pathway, the Nrf2/HO1 pathway, and the ferroportin (FPN) inhibition pathway.^[35]^ In terms of the specific mechanisms of antibacterial action, NCOA4 mediates the autophagic degradation of ferritin, thereby releasing iron ions and promoting lipid peroxidation reactions; the Nrf2/HO1 pathway, on the other hand, regulates antioxidant stress and further enhances the ferroptotic response.^[36]^ The substantial amount of ROS generated during the ferroptosis process can effectively facilitate the killing of bacteria by macrophages, which also confirms that ferroptosis synergizes with host immunity primarily in the aspect of cellular immunity.

### 5.2. “Iron death” of bacteria

Ferroptosis is an iron-dependent form of cell death initially identified in eukaryotic cells.^[14]^ It is characterized by the involvement of iron in causing the peroxidation of lipids in the cell membrane, leading to irreversible cellular damage and death.^[29]^ Although prokaryotes such as bacteria have a relatively simple structure and lack the complete molecular pathways associated with ferroptosis found in eukaryotic cells, they can exhibit a death phenomenon similar to ferroptosis, termed “ferroptosis-like cell death,”^[37]^ by regulating the levels of ferrous iron (Fe²^+^). Notably, research by Ruonan Ma has found that only ferrous iron (Fe²^+^) demonstrates significant bactericidal activity, while ferric iron (Fe³^+^) does not exhibit a similar effect, which is consistent with the phenomenon that most bacterial siderophores chelate and deliver ferric iron as a source of iron nutrition.^[37]^ The mechanism of bacterial “ferroptosis” may be beneficial for the development of targeted drugs that bypass host cells and act directly on bacteria to kill them, thus avoiding the impact of traditional ferroptosis reactions on host cells. Furthermore, the disruption of iron metabolism in combination with antibiotics may enhance the effectiveness of antibacterial treatment, particularly potentiating its application against multidrug-resistant bacteria.

### 5.3. Iron death inducer

Based on the aforementioned theory, ferroptosis inducers may become one of the new types of antimicrobial therapeutic agents. Ferroptosis inducers mainly induce an increase in intracellular iron by disrupting iron homeostasis and promoting iron metabolic disorders, ultimately triggering ferroptosis.^[38]^ The types of ferroptosis inducers mainly include Class I FIN, which inhibits the Xc-system; Class II FIN, which inhibits or degrades GPX4; Class III FIN, which consumes coenzyme Q10; and Class IV FIN, which induces lipid peroxidation through iron or polyunsaturated fatty acid (PUFA) overload. These 4 types are highly specific in inducing ferroptosis, meaning that they basically do not activate markers of other types of cell death during the process of inducing ferroptosis.^[39]^

At present, the clinical deployment of ferroptosis inducers is predominantly within the realms of oncology, specifically in antitumor treatments and radiotherapy, as well as chemotherapy. A notable example is the use of erastin, an inhibitor of the system xc−, which activates ferroptosis markers in xenografted tumors, particularly in cases of diffuse large B-cell lymphoma.^[40]^ In the context of radiotherapy, it has been observed that radiation can induce ferroptosis, and as such, ferroptosis inducers are being explored as potential radio-sensitizers to enhance the effectiveness of radiation therapy.^[41]^ Furthermore, in the field of immunotherapy, ferroptosis inducers are being investigated for their standalone use or in combination with other immunotherapeutic strategies to boost treatment outcomes.^[42]^ In the domain of antimicrobial treatment, the utilization of ferroptosis inducers remains in the experimental phase. Current research is primarily focused on several key areas, which include understanding the fundamental mechanisms by which these inducers engage with microbial pathogens, developing new compounds with enhanced potency and selectivity, and assessing the potential for combination therapies that could leverage the unique properties of ferroptosis to overcome AR:

Ferrous sulfate (FeSO_4_): Ferrous sulfate has been shown to rapidly eliminate *Staphylococcus aureus*, including methicillin-resistant *S aureus* (MRSA), by promoting ferroptosis-like bacterial cell death. In a mouse keratitis model, a hydrogel containing ferrous sulfate effectively cured *S aureus* infections and facilitated the rapid restoration of corneal health.^[43]^

Iron-containing nanoparticles: Iron-containing nanoparticles have demonstrated significant antimicrobial properties. An aqueous solution comprising ferrous ions and polysulfides (Fe(II)Sn_aq) has exhibited potent antimicrobial effects against *S aureus* by inducing ferroptosis-like cell death.^[6]^

Iron-loaded lipid nanoparticles (Fe-LNPs): These nanoparticles have been effective in inhibiting the growth of both *S aureus* and Escherichia coli, with their mechanism of action involving the induction of ferroptosis-like cell death.^[44]^

Fe(hinok)_3_: The Fe(hinok)_3_ complex, formed from the natural metal chelator hinokitiol and ferric iron, possesses high lipid solubility and nonpolarity, enabling it to penetrate bacterial cell membranes effectively. This complex bypasses bacterial iron homeostasis regulation, triggers the production of ROS, and ultimately leads to bacterial cell death.^[45]^

Iron-8-hydroxyquinoline complex (Fe(8-hq)_3_): The iron-8-hydroxyquinoline complex (Fe(8-hq)_**3**_) effectively disrupts the intracellular metal homeostasis of bacteria, exhibiting strong antimicrobial activity against MRSA. This iron complex not only functions as a metal chelator but also possesses inherent bactericidal properties.^[46]^

## 6. Iron death and multidrug resistance therapy

### 6.1. The difference between iron death and the antibacterial effect of traditional antibiotics

Ferroptosis distinguishes itself from the conventional antibiotic treatment of multidrug-resistant bacteria, primarily due to its unique mechanism of action. Traditional antibiotics typically combat infections by interfering with bacterial growth and replication, such as by inhibiting cell wall synthesis, protein synthesis, or DNA replication.^[47]^ However, multidrug-resistant bacteria have developed resistance to these antibiotics through various mechanisms, including genetic mutations and horizontal gene transfer, which has led to the increasing ineffectiveness of these traditional approaches.^[48]^

In contrast to traditional antibiotics, ferroptosis induces cell death by modulating iron metabolism, promoting the generation of ROS, and triggering lipid peroxidation.^[31]^ This mechanism has the potential to bypass traditional bacterial resistance mechanisms, as it primarily targets cellular iron metabolism and oxidative stress, rather than directly interfering with the fundamental physiological processes of bacteria.

The advantages of employing ferroptosis in antibacterial applications are manifold. Firstly, ferroptosis introduces a novel mechanism to tackle drug resistance, capable of circumventing many common resistance mechanisms. When used in conjunction with traditional antibiotics, ferroptosis inducers can potentiate antibacterial effects, thereby reducing the reliance on antibiotics and potentially decreasing the emergence of drug-resistant bacteria. Furthermore, the mechanism of ferroptosis is broadly applicable, unlike some specific antibiotics that are limited to targeting certain bacteria. Consequently, ferroptosis antibacterial therapy offers the distinct benefits of avoiding resistance development, reducing antibiotic usage, and exhibiting potential broad-spectrum efficacy.

### 6.2. Risks behind antimicrobial therapy for iron death

Ferroptosis inducers exert their antibacterial effects by increasing intracellular iron levels, which in turn trigger a ferroptotic response. This response leads to bacterial death through the generation of ROS via lipid peroxidation or by directly instigating a “ferroptosis-like reaction” in bacteria.^[37]^ In the context of normal human iron metabolism, the regulated flow and utilization of iron are vital for engaging in fundamental biological processes and disease prevention. Maintaining iron homeostasis is essential for health.^[10]^ Dietary heme and non-heme iron are absorbed in the intestine through distinct transport proteins.^[49]^ The protein Fpn1 on the basal side of the intestine mediates iron release and is regulated by the hepatic hormone hepcidin. Hepcidin controls iron egress from cells and its entry into the bloodstream by binding to Fpn1, promoting its internalization. Hepcidin expression is modulated by serum iron levels, iron storage, and inflammatory status.^[50]^ The predominant form of iron transport in serum is transferrin-bound iron, which undergoes endocytosis upon binding to cell surface Tfr1.^[51]^ Intracellular iron export across the cell membrane by Fpn1 is regulated by various factors, including oxidative stress and iron itself.^[50]^ iron regulatory proteins modulate iron levels by binding to iron-responsive elements on mRNA, thereby adjusting the synthesis of related proteins to preserve intracellular and systemic iron balance.^[52]^

Clinical application of ferroptosis inducers is not without potential side effects. These inducers may induce immune cell death, thereby diminishing the body’s immune defense capabilities.^[53]^ The accumulation of iron ions in the body could impair bone marrow function and affect hematopoiesis.^[54]^ Ferroptosis might also lead to hepatic and renal toxicity, resulting in organ functional damage.^[55]^ The treatment process could provoke severe weight loss and muscle wasting, presenting as cachexia.^[56]^ Moreover, the release of cellular contents due to ferroptosis may incite inflammatory responses, potentially promoting the formation of secondary tumors. This risk is escalated by the upregulation of CD274/PD-L1 and the infiltration of myeloid-derived suppressor cells (MDSCs), which may facilitate this process.^[57]^

The use of ferrous preparations that directly target bacteria to induce a ferroptosis-like reaction faces a graver challenge – iron poisoning. The total iron content in the human body is approximately 50 to 55 mg/kg body weight for adult males and 35 to 40 mg/kg for females. Iron is partitioned into functional iron, including hemoglobin iron constituting 67% of body iron, myoglobin iron accounting for 15%, and transferrin iron only 3 to 4 mg. The remainder is storage iron, typically around 1000 mg in males and 300 to 400 mg in females, comprising ferritin and hemosiderin. An average person requires 20 to 25 mg of iron daily for hematopoiesis, primarily sourced from senescent and lysed red blood cells.^[58]^ To sustain iron balance, an adult needs to ingest 1 to 1.5 mg of iron daily from food; pregnant and lactating women require 2 to 4 mg. Animal-derived food has a high iron absorption rate of up to 20%, whereas plant-derived food has a lower absorption rate of 1% to 7%.^[59]^ Iron intake exceeding 20 mg/kg body weight is generally considered low risk and does not necessitate special treatment. Intake between 20 and 60 mg/kg warrants home interventions, such as the use of syrup of ipecac and home surveillance. However, an intake surpassing 60 mg/kg is a medical emergency.^[60]^

Iron poisoning can manifest with digestive system symptoms, often initially presenting as vomiting, diarrhea, and other symptoms.^[61]^ The advanced stages of iron poisoning are marked by acute circulatory shock.^[62]^ The transformation of iron ions from the ferrous to the ferric state and the heightened inhibition of complex oxidative metabolism at the cellular level lead to increased lactate concentration, predisposing to metabolic acidosis.^[63]^ Free iron in the bloodstream inhibits thrombin’s action on fibrinogen and directly impacts the coagulation cascade, causing coagulopathy, and the capacity to generate thrombin is concurrently diminished.^[1]^

### 6.3. How to solve the risk of antibacterial treatment of iron death

Bacteria possess a more straightforward iron metabolism system compared to humans, characterized by the absence of complex iron regulatory mechanisms and a diminished capacity for antioxidant defense.^[64]^ While stringent control over the dosage of ferroptosis inducers or ferrous iron can mitigate toxic side effects, this strategy is hampered by the challenges associated with precise dosage control and the inherent risks involved. Polysulfides have the ability to react with cellular glutathione (GSH), leading to its depletion, and consequently, a significant reduction in bacteria’s antioxidant defense capabilities, rendering them more vulnerable to oxidative damage.^[6]^ Harnessing the properties of polysulfides can enhance the efficacy of ferroptosis inducers and minimize the quantities required.

In contrast to the aforementioned methods of dosage control or complex addition, nano-targeting techniques, such as magnetic nanoparticles (Fe_3_O_4_) and metal-organic frameworks (MOFs), may offer greater efficiency and safety. Nanometer-sized iron sulfide (nFeS) can more readily penetrate bacterial cells; its small size and distinctive surface properties facilitate more effective binding to or penetration of bacterial membranes.^[6]^ The thinner and less complex structure of bacterial cell membranes allows nFeS to enter and exert its function more efficiently, whereas the larger volume and intricate membrane structure of host cells offer greater resistance to nFeS invasion, thus reducing the impact on host cells.

Magnetic nanoparticles (Fe_3_O_4_), owing to their unique magnetic properties, hold significant potential in cancer treatment. They can be engineered as drug carriers and directed to tumor sites using magnetic fields, enabling precise drug delivery.^[65]^ Consequently, employing the properties of magnetic nanoparticles to transport ferroptosis inducers or ferrous iron directly to infected or bacterial cells, while bypassing normal human cells, represents a promising antibacterial strategy.

MOFs are a class of porous materials capable of acting as drug carriers, thereby increasing the load and targeting of therapeutic agents. MOFs can be loaded with ferroptosis inducers, such as erastin, to suppress tumor growth by promoting ferroptosis. Moreover, MOFs can generate heat through photothermal effects, effectively killing tumor cells.^[66]^

In summary, to prevent damage to normal human cells from ferroptosis inducers or ferrous iron, research efforts should be directed towards nanotechnology and drug carrier materials to achieve precise drug delivery. This approach can circumvent the toxic side effects associated with ferroptosis inducers or ferrous iron, allowing for the safe and effective utilization of ferroptosis in antibacterial treatment.

## 7. Novel antibacterial therapy based on iron death mechanism

Dai has developed an advanced ferroptosis bioheterojunction (F-bio-HJ), a composite structure integrating Fe_2_O_3_, Ti3C2-MXene, and glucose oxidase (GOx). This innovative system is designed to generate significant levels of ROS that specifically target the membranes of extracellular bacteria. Upon exposure to near-infrared (NIR) irradiation, the F-bio-HJ enhances the permeation of concurrently generated Fe2+/Fe3 + ions into bacteria, thereby inducing ferroptosis and leading to the demise of planktonic bacteria. Additionally, the F-bio-HJ facilitates the transport of Fe2 + into intracellular bacteria via the inward iron transporter ferroportin (FPN), promoting ferroptosis within these bacteria. As GOx consumes glucose, the F-bio-HJ triggers a protective starvation response that shields macrophages from ferroptosis by activating the adenosine 5’-monophosphate (AMP)-activated protein kinase (AMPK) pathway. In vivo studies have confirmed that the F-bio-HJ can promote the regeneration of diabetic infected skin without triggering ferroptosis in normal cells.^[67]^

Xue has explored a novel alkaline nanosword, enriched with strontium and calcium ferrite, fabricated through micro-arc oxidation and hydrothermal treatment methods. This nanosword is utilized in orthopedic implants to address postoperative infections and promote osteointegration. The ferrous ions released from the nanosword enter bacteria through the divalent iron transport system, inducing bacterial ferroptosis. This technology has shown success in mouse humeral transplantation models.^[68]^

Baohong Sun has introduced non-iron metal single-atom catalysts (Ir and Ru SACs) as a new bacterial strategy that emulates ferroptosis. These catalysts enhance the depletion of endogenous glutathione (GSH), thereby inhibiting the activity of the GPX4 enzyme and reducing thioredoxin reductase (TrxR) levels. They severely impair bacterial metabolic capacity and respiration, shifting aerobic respiration to anaerobic respiration, and induce severe oxidative stress, leading to damage to the bacterial genome and protein lipidation. Both ferroptosis-like inducers exhibit low toxicity in vitro and in vivo. The application of Ir and Ru SACs not only significantly accelerates the healing of MRSA-infected wounds and abscesses but also induces a specific pathogen immune memory response, reducing the risk of reinfection.^[69]^

In summary, the use of ferrous ions to directly elicit a ferroptosis-like reaction in bacteria may emerge as a key area of focus for future antibacterial treatment research. This method has the potential to bypass human cells, thus achieving antibacterial treatment without causing harm to the human body, provided that the targeting of ferrous ions is precisely ensured.

## 8. Limitations

Multidrug-resistant bacteria may already possess a more complex iron homeostasis regulatory mechanism, and diversified mechanisms help bacteria effectively obtain the iron needed for growth. At the same time, this makes the action of ferroptosis inducers in bacteria more complex, and bacteria may evade the action of ferroptosis inducers through various mechanisms.

The formation of biofilms may be one of the key factors in enhancing drug resistance. Biofilms provide a physical barrier for bacteria, preventing the penetration of foreign substances (including antimicrobial drugs and ferroptosis inducers).^[70]^ Bacteria within the biofilm are often in a state of reduced metabolism, known as persister cells, and these bacteria have a weaker response to many drugs, including ferroptosis inducers, in their low metabolic state.^[71]^ Due to the strong resistance of persister cells to oxidative stress, they are not easily killed by the ROS in the ferroptosis mechanism. In this way, the efficacy of ferroptosis inducers in bacteria within the biofilm is significantly reduced.^[72]^

Iron plays an important role in promoting the death of intracellular bacteria, but the specific molecular mechanisms still need further research.

It is currently unclear how macrophages precisely sense changes in the surrounding environment and regulate the pressure of ferroptosis.

The triggers and mediators of iron release that lead to bacterial ferroptosis have not yet been identified.

It is unknown whether ferroptosis exists in other microorganism pathogens beyond those already reported.

How to combat the critical infection stages of intracellular bacteria by regulating the availability of iron.

How to balance the damage to host cells and the elimination of intracellular pathogens when implementing ferroptosis strategies.

## 9. Conclusion

This review article discusses the novel programmed cell death mechanism of ferroptosis and its potential applications and challenges in the treatment of multidrug-resistant bacteria. Firstly, ferroptosis, which involves iron-catalyzed lipid peroxidation and the accumulation of ROS, can effectively kill bacteria.^[73]^ The ferroptosis-like cell death phenomenon in bacteria indicates that by modulating the levels of ferrous iron (Fe²^+^), bacteria also undergo a death pathway similar to ferroptosis, providing new therapeutic insights for targeting multidrug-resistant bacteria.^[74]^ Secondly, the advancement of nanotechnology offers possibilities for the precise delivery of ferroptosis inducers and the reduction of side effects. In experimental studies, several iron-loaded nanomaterials (such as Fe-LNPs, Fe(hinok)_**3**_, and Fe(8-hq)_**3**_) have demonstrated significant antibacterial activity, further proving the potential of nanotechnology in antimicrobial treatment. Thirdly, compared to traditional antibiotics, ferroptosis has a unique mechanism of action, directly killing bacteria by inducing cell membrane lipid peroxidation, rather than interfering with bacterial growth and replication processes. However, the potential side effects of ferroptosis inducers, such as immune cell death, bone marrow damage, and iron poisoning, need to be carefully controlled in clinical applications. To address the safety issues of ferroptosis-based antibacterial therapy, research should focus on using nanotechnology and drug carrier materials to achieve precise delivery. In particular, magnetic nanoparticles and MOFs show great potential in drug delivery and toxicity control. The future direction is to design new antimicrobial therapies based on the mechanism of ferroptosis, ensuring drug targeting and reducing damage to normal human cells to achieve safe and effective antimicrobial treatment. In summary, ferroptosis provides a new approach to solving the global medical challenge posed by multidrug-resistant bacteria, but its clinical application still requires further research to overcome related risks and achieve clinical translation. As demonstrated by researchers such as Dai and Xue, the application prospects based on nanotechnology and new catalysts are broad and are expected to play a key role in future antimicrobial therapies.

While ferroptosis presents a promising alternative for combating multidrug-resistant infections, significant challenges remain in its clinical translation. The complexity of iron metabolism regulation, potential host toxicity, and bacterial biofilm resistance pose substantial hurdles that require further investigation. Future research should focus on optimizing nanoparticle-based delivery systems to enhance targeting specificity, developing combination therapies to overcome biofilm barriers, and elucidating the molecular mechanisms governing bacterial ferroptosis. Advancements in these areas will be crucial for realizing the full therapeutic potential of ferroptosis-based antimicrobial strategies and addressing the growing global threat of AR.

## Author contributions

**Conceptualization:** Xiaohui Bi.

**Validation:** Gang Fu.

**Writing – review & editing:** Yonglang Chen, Lüwei Wen.
